# Determinants of time from symptom debut to dementia diagnosis in United Arab Emirates

**DOI:** 10.1192/bji.2025.10077

**Published:** 2026-11

**Authors:** Syed Fahad Javaid, Shamma Alkuwaiti, Nora Ibrahim, Fatima Alkaabi, Shaikha Al Houqani, Fadwa Al Mugaddam, Karim Abdel Aziz, Gabriel Andrade, Zubaida Shebani

**Affiliations:** 1 Department of Psychiatry, College of Medicine and Health Sciences, United Arab Emirates University, Al Ain, United Arab Emirates.; 2 College of Medicine and Health Sciences, United Arab Emirates University, Al Ain, United Arab Emirates; 3 Department of Psychology, College of Medicine, Ajman University, Ajman, United Arab Emirates; 4 Department of Psychology, Sultan Qaboos University, Muscat, Sultanate of Oman

**Keywords:** Dementia, DUS, mental health literacy, stigma and discrimination, United Arab Emirates

## Abstract

**Background:**

Although timely dementia diagnosis is a global healthcare priority, delays between symptom onset and formal recognition remain common, particularly in regions with limited awareness and high stigma. Such delays are associated with worse clinical outcomes, greater caregiver burden and increased healthcare costs. We report the first large-scale study of diagnostic delays in the United Arab Emirates (UAE) and the wider Middle East and North Africa, with findings relevant to other Gulf Cooperation Council countries, given the region’s unique demographic and cultural landscape.

**Aims:**

To investigate the duration between dementia symptom onset and formal diagnosis, and to identify demographic and clinical factors associated with prolonged delays.

**Method:**

We retrospectively analysed 825 patients diagnosed with dementia over 10 years at a major governmental hospital in Abu Dhabi. The duration of undiagnosed symptoms (DUS) was calculated and multivariable regression was performed, adjusting for age, gender, nationality, dementia subtype and presenting symptoms.

**Results:**

The mean DUS was 34.7 months (s.d. = 28.8), with a median of 24 months (interquartile range 12–48 months). Older adults (≥65 years) experienced longer delays than younger patients. Symptom profile strongly influenced DUS: patients presenting with memory complaints (forgetfulness) had the longest delays, whereas those with behavioural or psychiatric symptoms, such as agitation, hallucinations or disinhibition, were diagnosed earlier. In multivariable regression, older age and memory-related presentations remained independent predictors of longer DUS while gender, nationality and dementia subtype were not significant.

**Conclusions:**

Dementia diagnosis in the UAE is delayed by nearly 3 years, driven mainly by age and presenting symptoms. Reducing these delays through public awareness campaigns, clinician training and pilot primary care screening programmes could improve patient outcomes and reduce the growing health economic burden in the region.

Dementia is a broad term for neurodegenerative conditions that affect memory, thinking, language, other cognitive abilities and behaviour.^1^ It significantly interferes with an individual’s ability to perform daily activities, causing substantial distress to both patients and their families.^2^ Recent estimates indicate that over 55 million people worldwide are living with dementia,^3^ with projections suggesting a rise to 139 million by 2050.^4^ As global life expectancy increases, dementia is becoming a significant public health challenge, necessitating timely diagnosis and management.^5^ Recent estimates place the prevalence of dementia in the United Arab Emirates (UAE) at 1.33% while that across the Arab world is approximately 0.7%, with a substantial associated economic burden.^6^ The World Health Organization predicts that, in the Middle East and North Africa (MENA) region, the number of individuals living with dementia will reach 2.59 million by 2030, rising to over 6 million by 2050.^7^ In the UAE, demographic transitions and an increasing elderly population further emphasise the need for improved dementia care.

A major challenge in diagnosing dementia is the prolonged delay between symptom onset and formal medical recognition.^8,9^ Delayed diagnosis remains a persistent issue, influenced by various demographic, cultural and systemic factors.^10^ Studies indicate that delayed diagnosis is associated with poorer prognostic outcomes, increased caregiver burden and higher healthcare costs.^11^ Early detection enables better management, access to support services and potential interventions that may slow disease progression.^12^

Literature suggests that the time from symptom onset to diagnosis varies widely, depending on factors such as healthcare accessibility, cultural perceptions and health literacy.^13^ A significant contributor to delayed dementia diagnosis is the lack of structured screening programmes.^14^ Despite concerns regarding the accuracy of dementia screening tools and potential false positives, research consistently advocates for early screening due to the increasing prevalence of dementia and anticipated advancements in therapeutic interventions.^15^ Undiagnosed cognitive impairment can complicate the management of comorbidities, because patients with dementia frequently have serious coexisting conditions and take multiple medications.^16^ Without proper cognitive assessment, these conditions may be mismanaged, affecting treatment adherence and overall patient outcomes.^15^

In the UAE and countries with similar cultural backgrounds, the stigma associated with cognitive decline remains a major obstacle.^17,18^ In societies where mental health issues are not openly discussed, families often underestimate early symptoms, attributing these to normal ageing rather than recognising them as signs of neurodegeneration.^19^ Additionally, limited awareness among healthcare providers regarding mild cognitive impairment, lack of confidence in diagnosing patients with dementia and the absence of standardised diagnostic protocols further exacerbate delays.^19^

To our knowledge, this is the first large-scale study to examine diagnostic delay in dementia within the MENA region. There is limited evidence available in this regard, primarily from countries with well-developed dementia care settings.^14,20–22^ The UAE’s demographic profile, characterised by a rapidly ageing population and a large expatriate community, provides valuable insights not only nationally but also across other Gulf Cooperation Council (GCC) countries with similar healthcare challenges. This study seeks to analyse the average duration of cognitive and behavioural symptoms before a formal dementia diagnosis within a UAE-specific population. We also report the symptom profile of dementia patients in the UAE, highlighting common presenting issues and their impact on the duration of undiagnosed dementia. By investigating demographic and systemic contributors, including age, gender and presenting symptoms, this research provides critical insights for shaping future dementia care policies in the UAE. Furthermore, these findings contribute to the global discourse on dementia diagnosis by addressing the unique challenges faced in the UAE healthcare system, and by providing region-specific strategies to enhance early detection and intervention efforts for dementia.

## Method

This study was conducted at the Behavioural Sciences Institute (BSI) of Al Ain Hospital, Al Ain City, UAE, one of the largest governmental hospitals in the eastern region of Abu Dhabi (the largest of the seven emirates of UAE). Its psychiatric services are well developed and serve the population of Al Ain City and surrounding towns, which numbers over 600 000.^23^ Ethical approval for this study was obtained from the Al Ain Hospital Research Ethics Governance Committee (approval no. AAHEC-11-20-030). Consent to participate was not applicable in this retrospective data-based study.

### Study design and participants

Data were extracted retrospectively from patient electronic medical records for subjects diagnosed with dementia and presenting to the BSI over a 10-year period (2010–2019). Patient confidentiality was safeguarded by extracting only de-identified data from the electronic medical records. No personal identifiers were collected, and analyses were conducted on anonymised data-sets in accordance with ethics committee approval. The investigators then identified and extracted the relevant data needed to conduct their study.

We included male and female subjects over the age of 18 years, of all nationalities, with a principal documented diagnosis of dementia and who were registered with BSI. Subjects were excluded if they had a diagnosis of intellectual disability, traumatic brain injury or cerebrovascular event, or a concurrent mental disorder, as a principal diagnosis. Due to the retrospective nature of the study, the exact number of excluded cases could not be reliably quantified (see Supplementary Fig. 1, Additional file 1). From the electronic medical records, data were obtained for the following: demographics (age at the time of diagnosis, gender and nationality); presenting symptoms; principal diagnosis; and the duration of undiagnosed symptoms of dementia (DUS), i.e. the number of months between the participant’s first cognitive/behavioural symptoms and a formal diagnosis of dementia. Symptom onset was determined from caregiver-reported information, as documented in the electronic medical records at the time of clinical assessment. Documentation of symptom onset was narrative and not standardised, because caregiver accounts were recorded in routine clinical notes rather than in structured forms. Cognitive test scores, including the Mini-Mental State Examination (MMSE) and Montreal Cognitive Assessment (MoCA), were extracted from electronic medical records when available; however, documentation was inconsistent and the data were too sparse for inclusion in the analyses.

### Measures

For the diagnosis of dementia, ICD-10 criteria were used.^24^ ICD-10 describes dementia as a syndrome resulting from a brain disease, typically chronic or progressive, characterised by disturbances in various higher cortical functions such as memory, thinking, orientation, comprehension, calculation, learning ability, language and judgement, without affecting consciousness.^24^ Dementia cases were coded as ‘unspecified’ when documentation did not permit a reliable subtype classification – for example, due to the absence of biomarker confirmation, lack of advanced neuroimaging or insufficient clinical detail in the records. Neuropsychiatric symptoms were evaluated as described in the Neuropsychiatric Inventory Questionnaire (NPI-Q).^25^ This covers the following symptoms: delusion, hallucination, elation/euphoria, agitation/aggression, disinhibition, irritability/lability, depression/dysphoria, anxiety, apathy/indifference, appetite/eating, night-time behaviour and aberrant motor behaviour.

### Data tools

The study involved extraction of patient data from medical records by members of the research team, using a nine-item questionnaire (see Additional file 2), for each patient record that matched the aforementioned inclusion criteria. The questionnaire collected demographic information, including age at the time of diagnosis, gender and nationality. It also collected data about the diagnosis, presenting symptoms and their onset.

### Statistical analysis

*A priori* power analysis was conducted using G*Power 3.1.9.4 software for Windows (Heinrich Heine University Düsseldorf, Düsseldorf, Germany; https://www.psychologie.hhu.de/arbeitsgruppen/allgemeine-psychologie-und-arbeitspsychologie/gpower).^26^ For analysis of variance (ANOVA), we specified 13 groups (representing the 13 symptoms), a medium effect size, a desired power of 0.8 and an alpha level of 0.05. The analysis indicated that a total sample size of 288 participants would be sufficient to detect a medium effect with adequate power. Our sample size (*N* = 825) far exceeds this requirement, thereby ensuring robust statistical validity. Additionally, we performed a power analysis for a two-tailed *t*-test with a medium effect size, alpha of 0.05 and power of 0.8, which yielded a required sample size of 128. Again, with 825 participants, our sample size was more than adequate for this analysis too.

Values of the data analysed are expressed as means ± s.d. Where relevant, *t*-tests were carried out and *P*-values equal to or less than 0.05 were considered statistically significant. The assumption of homogeneity of variance for a two-tailed independent *t*-test was assessed using Levene’s test. In the case where the assumption of homogeneity was not met after conducting Levene’s test, Welch’s test was performed. A post hoc Tukey’s test was conducted to determine the statistical significance of the differences in DUS between initial symptoms.

A multiple linear regression analysis was conducted to examine the association between demographic factors, dementia types and initial symptoms with the dependent outcome (DUS score). The model included predictors such as age at diagnosis, gender, nationality (Emirati versus non-Emirati), dementia subtype and initial symptom clusters (e.g. agitation, hallucinations, depression, motor disturbance). Standardised estimates, standard errors, confidence intervals and significance levels were calculated, and overall model fit was evaluated using *R*, *R* ^2^, *F*-statistics and *P*-values.

## Results

### Demographic and clinical characteristics

Eight hundred and twenty-five (825) patients were identified using the inclusion criteria. The mean age of diagnosis was 78 years (s.d. = 11.1). The range of afflictions spanned from the youngest patient, at 39 years, to the oldest, at 117 years. The demographic characteristics of the sample are presented in Table 1.

**Table 1:** Demographic characteristics of the study sample (*N* = 825) Table 1 long description.

Characteristics	*n*	Percentage of total
Gender		
Male	383	46.4
Female	442	53.6
Age group (years)		
<70	164	19
70+	661	80
Nationality		
Emirati	518	62.8
Non-Emirati	307	37.2

The most frequent type of dementia diagnosed was unspecified dementia (42.1%), followed by Alzheimer’s disease (40.6%). Table 2 depicts the distribution of different types of dementia among our participants.

**Table 2:** Types of dementia in the study sample (*N* = 825) Table 2 long description.

Type of dementia	*n*	Percentage of total
Alzheimer’s dementia	335	40.6
Vascular dementia	82	9.9
Unspecified	354	42.9
Frontotemporal dementia	5	0.6
Parkinson’s disease dementia	32	3.9
Lewy body dementia	4	0.5
Mixed dementia	11	1.3
Posterior cortical atrophy	2	0.2

### DUS

The mean DUS was 2.89 years (34.7 months; s.d. = 28.8), with a median of 24 months (interquartile range (IQR) 12–48). A small number of extreme outliers were identified (>121 months, >3 s.d. above the mean), but sensitivity analyses excluding these cases (*n* = 822) yielded unchanged medians (24 months, IQR 12–48) and minimal changes in means (34.2 *v.* 34.6), confirming the robustness of the findings. These findings are presented in Supplementary Table 1 (Additional file 3).

**Fig. 1:**
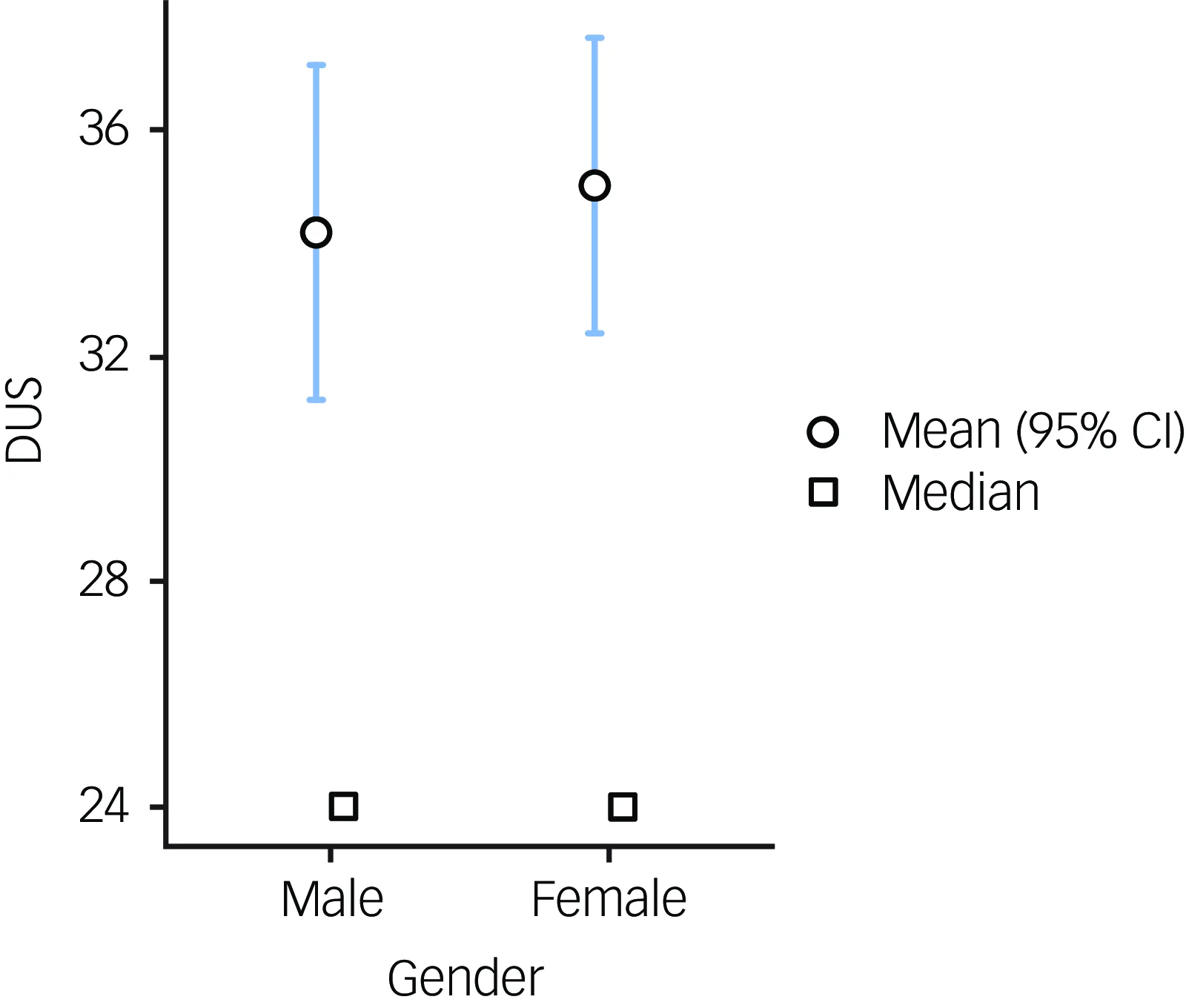
Gender comparison regarding the duration of undiagnosed symptoms (DUS) of dementia.

The mean DUS among female study participants was 35 months (s.d. = 28) compared with 34 months (s.d. = 29.6) in male participants. Both males and females had a median DUS of 24 months (IQR 12–48). A two-tailed independent *t*-test was conducted to assess the effect of gender on DUS. No significant difference in DUS was found between males and females (*t*(823) = −0.41, *P* = 0.69, Cohen’s *d* = −0.03), as depicted in Fig. 1. Given the large sample size (*N* = 825), the normality assumption was not necessary. The homogeneity of variance assumption was met following application of Levene’s test (*F*(1, 823) = 0.01, *P* = 0.95).

**Fig. 2:**
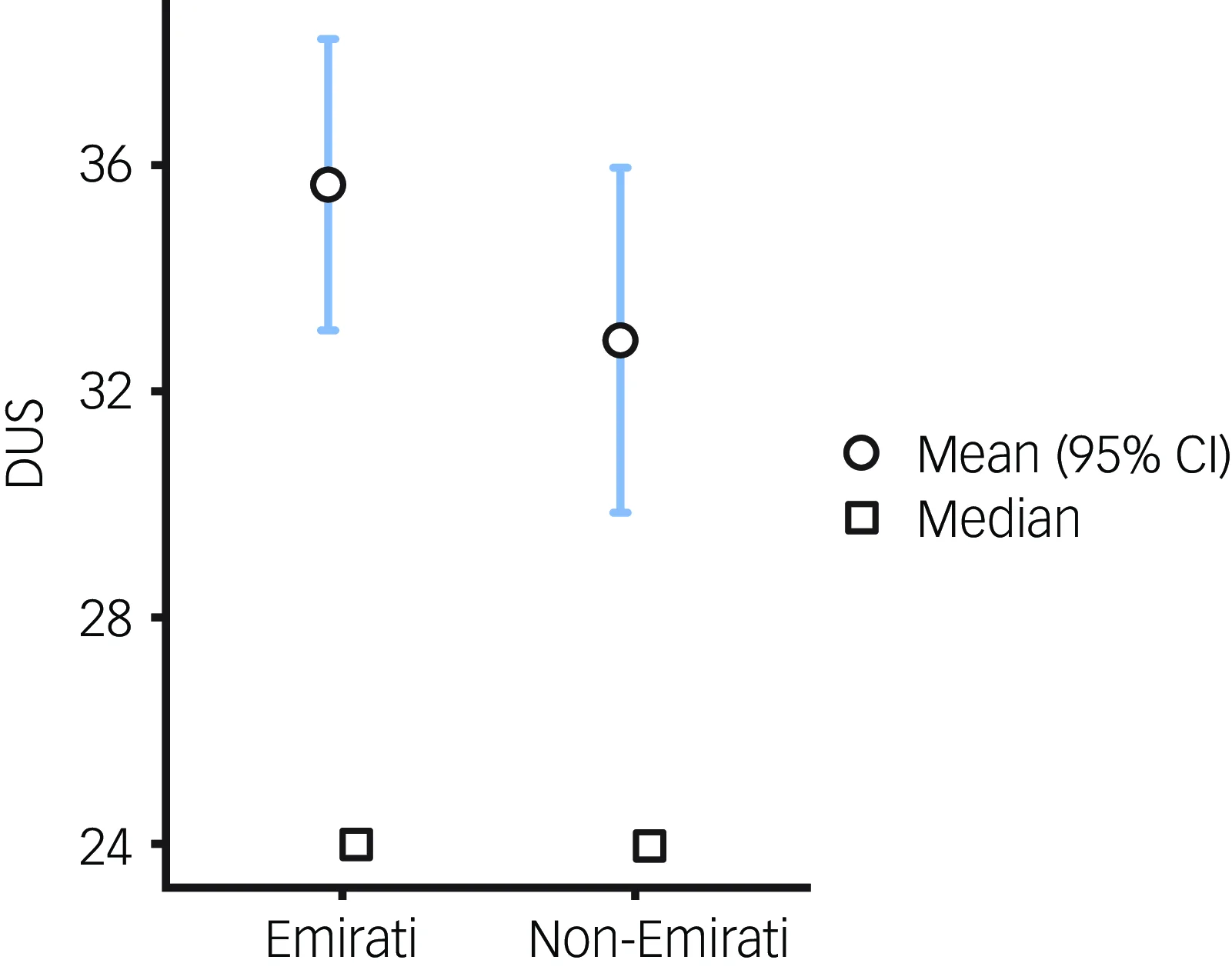
Nationality group comparison in the duration of undiagnosed symptoms (DUS) of dementia between Emirati and non-Emirati study participants.

The mean DUS among Emirati participants was 35.6 months (s.d. = 29.7), compared with 33 months (s.d. = 27) in non-Emiratis. The median DUS (24 months, IQR 12–48) was similar between Emirati and non-Emirati participants. A two-tailed independent *t*-test was conducted to assess the effect of the nationality group on DUS; there was no significant difference between Emiratis and non-Emiratis in DUS (*t*(823) = 1.33, *P* = 0.19, Cohen’s *d* = 0.10), as shown in Fig. 2. Given the large sample size (*N* = 825), the normality assumption was not necessary. The homogeneity of variance assumption was met following application of Levene’s test (*F*(1, 823) = 1.13, *P* = 0.29).

**Fig. 3:**
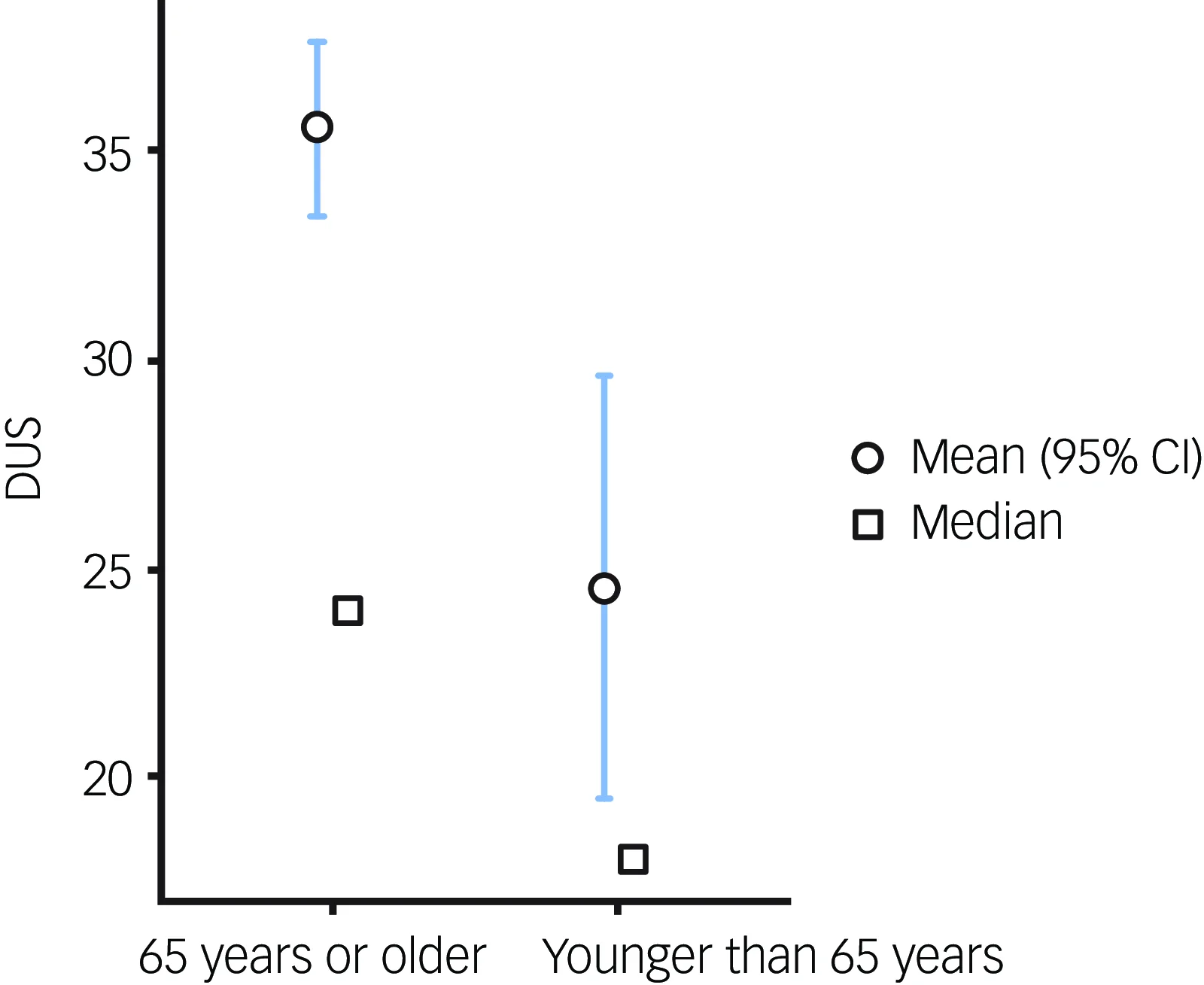
Age group comparison of the duration of undiagnosed symptoms (DUS) of dementia between participants aged 65 years and older and those under 65 years.

The cohort was divided into two groups: those under 65 years old and those 65 years or older at the time of diagnosis, in line with the traditional cut-off used in literature.^27^ The mean DUS among study participants under 65 years old at the time of diagnosis was 24.5 months (s.d. = 21.4), compared with 35.6 months (s.d. = 29.2) among those older than 65. Those under 65 years had a shorter median DUS of 18 months (IQR 12–30), while those aged 65 and above had a median of 24 months (IQR 12–48). The assumption of homogeneity of variance for a two-tailed independent *t*-test to assess the effect of age group (younger than 65 years, 65 or older) was not met following the application of Levene’s test (*F*(1, 823) = 16.8, *P* < 0.001; consequently, Welch’s test was conducted to assess the effect of age group on DUS. There was a significant effect on age group (*t*(92.9) = 3.98, *P* < 0.001), with an effect size of *d* = 0.43, where individuals 65 years or older had longer DUS, as shown in Fig. 3.

One-way ANOVA was conducted to assess the effect of initial symptoms (forgetfulness, agitation/aggression, delusions, depression/dysphoria, irritability/lability, motor disturbance, appetite/eating, hallucinations, apathy/indifference, anxiety, night-time behaviours, elation/euphoria and disinhibition) on DUS. Forgetfulness (60.2%) was the most common presenting symptom. Table 3 depicts DUS according to symptom group in our sample.

**Table 3:** Duration of undiagnosed symptoms (DUS) according to initial symptoms (*N* = 825) Table 3 long description.

Initial symptoms	*n*	Mean DUS (months)	s.d.	Median (IQR)
Forgetfulness	497	42.9	29.14	36.0 (24.0–60.0)
Agitation/aggression	94	23.19	25.06	18.0 (6.0–24.0)
Depression/dysphoria	35	27.66	19.94	24.0 (18.0–36.0)
Delusions	11	24	29.23	12.0 (4.5–36.0)
Irritability/lability	49	26.65	21.61	18.0 (12.0–36.0)
Motor disturbance	43	19.93	24.23	12.0 (6.0–24.0)
Appetite/eating	19	16.37	17.42	6.0 (4.0–24.0)
Hallucinations	33	10.24	12.37	8.0 (5.0–12.0)
Apathy/indifference	8	44.25	39.66	36.0 (12.0–57.0)
Anxiety	8	15.13	8.84	12.0 (12.0–19.5)
Night-time behaviours	20	24.90	27.71	12.0 (6.0–39.0)
Disinhibition	8	8.38	8.68	4.0 (2.5–13.5)
Elation/euphoria	0	NA	NA	NA

IQR, interquartile range.

High standard deviations in some categories reflect small subgroup size (e.g. apathy/indifference, *n* = 8) and the potential influence of outliers.

There was a significant effect of initial symptoms on DUS (*F*(11, 69.2) = 22, *P* < 0.001, *η* ^2^ = 0.14). A post hoc Tukey’s test was conducted to determine the statistical significance of the differences between each pair of initial symptoms. There was a statistically significant mean difference in DUS between forgetfulness and agitation/aggression (19.7, *P* < 0.001), between forgetfulness and irritability/lability (16.24, *P* < 0.003), between forgetfulness and motor disturbance (22.97, *P* < 0.001), between forgetfulness and appetite/eating (26.53, *P* = 0.002), between forgetfulness and hallucinations (32.65, *P* < 0.001) and between forgetfulness and disinhibition (34.52, *P* < 0.017).

The overall regression model was statistically significant (*R* = 0.509, *R* ^2^ = 0.259, *F*(21, 803) = 13.4, *P* < 0.001), explaining about 25.9% of variance in the dependent outcome. Age at diagnosis was a significant positive predictor (*P* < 0.001). Several initial symptom presentations showed negative associations when compared with the reference category: forgetfulness, including agitation/aggression; delusions; irritability/lability; motor disturbance; appetite/eating changes; hallucinations; anxiety, night-time behaviours; disinhibition; and depression/dysphoria (all *P* < 0.05). In contrast, apathy/indifference was not significant. Regarding gender, females did not differ significantly from males. Similarly, Emirati status showed no effect when comparing non-Emirati individuals with Emiratis. For dementia subtypes (reference, Alzheimer’s disease), Parkinson’s disease dementia demonstrated a significant negative association (*P* = 0.045). Other subtypes, including vascular dementia, Lewy body dementia, frontotemporal dementia, mixed dementia and posterior cortical atrophy, did not differ significantly from Alzheimer’s disease in this model. Full regression results are provided in Supplementary Table 2 (Additional file 4).

## Discussion

This study investigated the average duration of cognitive, behavioural and neuropsychiatric symptoms prior to participants receiving a formal dementia diagnosis. Additionally, we report the initial symptom profile of dementia patients in the UAE and its association with DUS.

Across the sample, the mean duration between symptom onset and confirmed diagnosis of dementia was 2.89 years, or 34.7 months, indicating a substantial delay in diagnosis and considerable variability among individuals. The sensitivity analysis excluding extreme outliers confirmed the robustness of these findings, showing that the overall conclusions are not driven by a small number of atypical cases. Several rigorously conducted studies have reported the mean duration from symptom onset to diagnosis of dementia.^14,20–22^ The mean diagnostic delay of nearly 3 years in our cohort is notably longer than that reported in high-income countries, such as Norway, where mean delay was approximately 20 months.^14^ This disparity highlights the impact of health system and sociocultural factors in the UAE and the wider region, underscoring the need for tailored strategies to narrow the diagnostic gap.

A high proportion of cases (42.1%) were classified as ‘unspecified dementia’. This reflects challenges in subtype classification in routine clinical practice in the region, where biomarker testing and advanced imaging are not always available. Similar findings have been reported by El-Metwally and colleagues in Middle Eastern cohorts, underscoring the need for improved diagnostic infrastructure and more consistent application of subtype criteria.^18^ Our findings highlight the under-recognition of dementia in its early stages and the variability of diagnostic pathways, underscoring the need for greater public awareness and primary care screening. Practical region-specific strategies include piloting of brief cognitive screening tools, embedding dementia messages into national health campaigns and providing targeted training for general practitioners and community health workers. These represent feasible interventions within existing health system structures in the UAE and GCC region to reduce diagnostic delays.

Delays in identifying and diagnosing dementia in the UAE and the broader Middle Eastern context are influenced by a confluence of cultural, systemic and clinical factors.^19^ Cultural attitudes towards ageing and cognitive decline play a pivotal role in postponing dementia diagnoses. In Middle Eastern societies, public knowledge gaps and stigma, including misattribution of early cognitive symptoms to ‘normal ageing’, are well documented.^28,29^ This perception leads to the normalisation of early dementia symptoms, resulting in postponed medical consultations.^28^ Furthermore, the stigma associated with mental health conditions can discourage families from seeking timely medical advice due to fears of social repercussions. While mental health and dementia issues are stigmatised at the global level,^30^ unique cultural challenges within Middle Eastern countries, such as the UAE, should also be considered in relation to the results of this current study. These include pervasive and negative culturally held views surrounding mental illness, with a strong belief that such experiences are the result of God’s punishment, evil spirits or black magic.^31^ Stigma and cultural context may differentially influence help-seeking among Emirati and expatriate families. For expatriate communities, language barriers and difficulty in navigating services can hinder early presentation. Furthermore, the lack of standardised, culturally adapted diagnostic tools in Arabic, coupled with high illiteracy rates among older adults, hinder accurate and timely detection.^32^ These findings suggest that sociocultural norms, language and health literacy shape diagnostic pathways differently across groups in the UAE and wider GCC, helping to explain the longer delays associated with memory-led presentations.

In this study, individuals over the age of 65 years were statistically more likely to experience a longer DUS compared with those under 65. Age-related biases and misconceptions significantly impact the timeliness of dementia diagnoses.^4^ Older adults may encounter delays due to the normalisation of cognitive decline with ageing, while younger individuals face challenges stemming from the atypical nature of their symptoms.^33^ A deficit in health literacy concerning conditions such as dementia remains a significant challenge in the UAE.^34^ However, considering that there was no statistically significant difference between Emirati and non-Emirati participants regarding DUS in this study, it can be inferred that the issue of stigma and limited health literacy is not confined to the UAE but is a global concern across cultures, indicating the universal and indiscriminate nature of dementia as a neurodegenerative condition.^35^

Our study revealed no statistically significant difference between males and females in regard to DUS. This is intriguing, because gender is a significant determinant in accessing mental health services, characterised by a multidimensional disparity involving social norms, help-seeking behaviours, healthcare biases and systemic inequalities.^36^ Previous research has identified female gender as being associated with delays in the referral and diagnosis of dementia, reflecting an implicit bias that links cognitive decline more strongly with male behavioural presentation.^37^

The observed lack of statistically significant differences in DUS between nationalities and genders may indicate underlying issues in the UAE health services when addressing dementia, given that the common denominator across the sample was the Emirati health service accessed by the study participants. The knowledge of clinicians has been noted in other literature as being vital for ensuring timely referrals.^38^ There is a perceived lack of understanding within UAE health services regarding the symptoms and challenges of living with dementia.^18^ It can be argued that, alongside the general population, frontline health professionals may also need increased awareness about dementia. Public health bodies in the UAE should also aim to promote campaigns that destigmatise dementia and highlight the importance of early help-seeking behaviours.

Concerning presenting symptoms, the sample was categorised into various groups (forgetfulness, agitation/aggression, delusions, anxiety, etc.). The results revealed significant mean differences in DUS between forgetfulness and a variety of other presenting symptoms, including aggression, lability, motor disturbance, appetite issues, hallucinations and disinhibition. The most frequent clinical presentation of dementia is progressive loss of memory.^39^ While most individuals can recognise forgetfulness as a core symptom of dementia, other symptoms may be more likely to elicit a response from key stakeholders. These secondary presenting symptoms may diverge significantly from common perceptions of dementia, prompting more urgent reactions from both family members and clinicians. For instance, hallucinations and motor disturbances are crucial components of Lewy body dementia^40^ yet they can have a far greater impact on daily life, particularly in the early stages, than forgetfulness.^41^ As previously mentioned, this may closely correlate with the level of dementia literacy among the public and professionals alike.

To further explore these associations, we conducted a multivariable regression adjusting for age, gender, nationality, dementia subtype and presenting symptoms. In this adjusted model, older age and memory-related presentations remained significant independent predictors of longer diagnostic delays. Gender and nationality were not significant predictors once other factors were accounted for, consistent with the univariate results. Dementia subtype was not independently associated with DUS, although the large proportion of unspecified cases may have limited the statistical power. These findings reinforce the observation that age and symptom profile, rather than demographic factors, exert the strongest influence on diagnostic delay.

Importantly, the persistence of symptom-related effects in the adjusted model highlights how clinical presentation shapes pathways to diagnosis. Forgetfulness, the most common presenting feature, was independently associated with prolonged delays whereas behavioural and psychiatric symptoms (e.g. hallucinations, agitation) prompted earlier clinical recognition. This suggests that both families and clinicians may normalise memory loss as part of ageing, while disruptive behavioural features trigger more urgent help-seeking and referral.

This is the first large-scale study in the region to quantify delays in dementia diagnosis and explore symptom profiles. The large sample size (*N* = 825) and the use of real-world clinical data enhanced the ecological validity and relevance of the findings for healthcare planning. However, limitations exist; the retrospective design restricts symptom onset accuracy, relying on caregiver-reported data in medical records. This may introduce recall bias, because caregivers could confuse early symptoms with normal ageing or report them inaccurately. Variability in recording caregiver symptom reports, due to non-standardised documentation, may have impacted accuracy. Prospective studies with standardised symptom logs could yield more precise diagnostic delay estimates. Although we examined demographic and symptom-related predictors, unmeasured confounders such as education, comorbidities and socioeconomic factors were not consistently documented in the medical records and could not be included, thereby limiting our ability to account fully for factors contributing to delays in dementia diagnosis. Cognitive test scores (MMSE, MoCA) were inconsistently documented in the retrospective data-set and could not be analysed, limiting assessment of the link between cognitive severity and diagnostic delay, and these should be addressed in future prospective studies. Although our study focuses on patient factors, system-level barriers may also contribute to delayed dementia diagnosis in the UAE.^42^ Despite a large sample size, this study relied on a single specialist centre, which may have limited its generalisability to other healthcare settings. The UAE’s mixed healthcare system, comprising both public and private providers, features distinct referral pathways, resources and costs. Research in the Arab region highlights financial, administrative and organisational barriers to accessing specialist or high-quality care.^43^ These structural differences were not captured and are a further potential limitation. The lack of qualitative data constrained insight into caregivers’ perspectives and lived experiences behind the delayed diagnosis, because symptom classification using NPI-Q might have excluded subtle or culturally framed symptoms not captured in inventories. Future mixed-methods research, including qualitative interviews with families and clinicians, will be important in exploring perceptions of ‘forgetfulness’ and help-seeking norms.

This study reveals a delay of nearly 3 years between symptom onset and dementia diagnosis among older UAE adults. Gender and nationality did not affect delays, but older age and symptoms did. It highlights gaps such as low dementia literacy, stigma and lack of structured screening pathways in the UAE. This study underscores the importance of examining global issues through a local lens to identify potential improvements. Reducing diagnostic delays can also lessen the health economic burden of dementia in the UAE and the Arab region. Future research should incorporate qualitative methods to explore personal, cultural and healthcare system-level barriers from the perspectives of both families and clinicians.

## Supporting information

Javaid et al. supplementary material 1 Javaid et al. supplementary material

Javaid et al. supplementary material 2 Javaid et al. supplementary material

Javaid et al. supplementary material 3 Javaid et al. supplementary material

Javaid et al. supplementary material 4 Javaid et al. supplementary material

## Data Availability

The data that support the findings of this study are available from the corresponding author, S.F.J., on reasonable request.

## Supplementary material

The supplementary material for this article is available online at https://doi.org/10.1192/bji.2025.10077.

## Table 1 Long description

The table presents demographic characteristics of a study sample of 825 patients. It has three columns: Characteristics, n, and Percentage of total. The table is divided into three main categories: Gender, Age group, and Nationality. Row 1: Gender, Male, 383, 46.4 percent. Row 2: Gender, Female, 442, 53.6 percent. Row 3: Age group, less than 70 years, 164, 19 percent. Row 4: Age group, 70 years or older, 661, 80 percent. Row 5: Nationality, Emirati, 518, 62.8 percent. Row 6: Nationality, Non-Emirati, 307, 37.2 percent.

Navigate back to Table 1.

## Table 2 Long description

A table titled 'Types of dementia in the study sample (N = 825)' with two columns and eight rows. The first column is labeled 'Type of dementia' and the second column is labeled 'Percentage of total'. The table lists the following types of dementia and their respective percentages: Alzheimer's dementia (40.6 percent), Vascular dementia (9.9 percent), Unspecified (42.9 percent), Frontotemporal dementia (0.6 percent), Parkinson's disease dementia (3.9 percent), Lewy body dementia (0.5 percent), Mixed dementia (1.3 percent), and Posterior cortical atrophy (0.2 percent).

Navigate back to Table 2.

## Table 3 Long description

The table presents data on the duration of undiagnosed symptoms (DUS) according to initial symptoms in a sample of 825 individuals. It includes 13 rows and 5 columns. The columns are labeled Initial symptoms, n, Mean DUS (months), s.d., and Median (IQR). The row labels include various initial symptoms such as Forgetfulness, Agitation/aggression, Depression/dysphoria, Delusions, Irritability/lability, Motor disturbance, Appetite/eating, Hallucinations, Apathy/indifference, Anxiety, Night-time behaviours, Disinhibition, and Elation/euphoria. Each row provides the number of individuals (n), the mean duration of undiagnosed symptoms in months, the standard deviation (s.d.), and the median duration with the interquartile range (IQR). Notable trends include Forgetfulness having the highest mean DUS at 42.9 months and the largest sample size of 497 individuals, while Disinhibition has the lowest mean DUS at 8.38 months and a sample size of 8 individuals. Elation/euphoria is not present in the data.

Navigate back to Table 3.

## References

[1] American Psychiatric Association. Diagnostic and Statistical Manual of Mental Disorders: DSM-5TM 5th ed. American Psychiatric Publishing, Inc., 2013.

[2] Livingston G , Huntley J , Sommerlad A , Ames D , Ballard C , Banerjee S , et al. Dementia prevention, intervention, and care: 2020 report of the Lancet Commission. Lancet 2020; 396: 413–46. 10.1016/S0140-6736(20)30367-6 PMC7392084 32738937

[3] Schwarzinger M , Dufouil C. Forecasting the prevalence of dementia. Lancet Public Health 2022; 7: e94–5. 10.1016/S2468-2667(21)00277-2 34998486

[4] Lai M , Jeon Y-H , McKenzie H , Withall A. Journey to diagnosis of young-onset dementia: a qualitative study of people with young-onset dementia and their family caregivers in Australia. Dementia (London) 2023; 22: 1097–114. 10.1177/14713012231173013 PMC10262327 37126513

[5] Nichols E , Steinmetz JD , Vollset SE , Fukutaki K , Chalek J , Abd-Allah F , et al. Estimation of the global prevalence of dementia in 2019 and forecasted prevalence in 2050: an analysis for the Global Burden of Disease Study 2019. Lancet Public Health 2022; 7: e105–25. 10.1016/S2468-2667(21)00249-8 PMC8810394 34998485

[6] Qassem T , Itani L , Nasr W , Al-Ayyat D , Javaid SF , Al-Sinawi H. Prevalence and economic burden of dementia in the Arab world. BJPsych Open 2023; 9: e126. 10.1192/bjo.2023.517 PMC10375885 37439065

[7] World Health Organization. Dementia: A Public Health Priority. WHO, 2012 (https://www.who.int/publications/i/item/dementia-a-public-health-priority).

[8] Bamford C , Lamont S , Eccles M , Robinson L , May C , Bond J. Disclosing a diagnosis of dementia: a systematic review. Int J Geriatr Psychiatry 2004; 19: 151–69. 10.1002/gps.1050 14758581

[9] Paterson NE , Pond D. P1-047: the barriers to the early diagnosis of dementia and diagnostic disclosure in primary care. Alzheimers Dement 2009; 5: P185.

[10] Prince M , Ali G-C , Guerchet M , Prina AM , Albanese E , Wu Y-T. Recent global trends in the prevalence and incidence of dementia, and survival with dementia. Alzheimers Res Ther 2016; 8: 23. 10.1186/s13195-016-0188-8 PMC4967299 27473681

[11] Wilson DM , Cookson MR , Bosch LVD , Zetterberg H , Holtzman DM , Dewachter I. Hallmarks of neurodegenerative diseases. Cell 2023; 186: 693–714. 10.1016/j.cell.2022.12.032 36803602

[12] Parker M , Barlow S , Hoe J , Aitken L. Persistent barriers and facilitators to seeking help for a dementia diagnosis: a systematic review of 30 years of the perspectives of carers and people with dementia. Int Psychogeriatr 2020; 32: 611–34. 10.1017/S1041610219002229 32024558

[13] Bernstein Sideman A , Al-Rousan T , Tsoy E , Piña Escudero SD , Pintado-Caipa M , Kanjanapong S , et al. Facilitators and barriers to dementia assessment and diagnosis: perspectives from dementia experts within a global health context. Front Neurol 2022; 13: 769360. 10.3389/fneur.2022.769360 PMC8997042 35418934

[14] Helvik A-S , Engedal K , Šaltytė Benth J , Selbæk G. Time from symptom debut to dementia assessment by the specialist healthcare service in Norway. Dement Geriatr Cogn Dis Extra 2018; 8: 117–27. 10.1159/000487233 PMC5921216 29706988

[15] Milne A. Dementia screening and early diagnosis: the case for and against. Health Risk Soc 2010; 12: 65–76.

[16] Browne J , Edwards DA , Rhodes KM , Brimicombe DJ , Payne RA. Association of comorbidity and health service usage among patients with dementia in the UK: a population-based study. BMJ Open 2017; 7: e012546. 10.1136/bmjopen-2016-012546 PMC5353300 28279992

[17] Javaid SF , Al-Zahmi A , Abbas M. Carer empowerment is key to reduce dementia care inequalities in the Middle East. Int J Environ Res Public Health 2021; 18: 4378. 10.3390/ijerph18084378 PMC8074373 33924161

[18] El-Metwally A , Toivola P , Al-Rashidi M , Nooruddin S , Jawed M , AlKanhal R , et al. Epidemiology of Alzheimer’s disease and dementia in Arab countries: a systematic review. Behav Neurol 2019; 2019: 3935943. 10.1155/2019/3935943 PMC6854962 31772681

[19] Aminzadeh F , Molnar FJ , Dalziel WB , Ayotte D. A review of barriers and enablers to diagnosis and management of persons with dementia in primary care. Can Geriatr J 2012; 15: 85–94. 10.5770/cgj.15.42 PMC3521322 23259021

[20] Zhao M , Lv X , Tuerxun M , He J , Luo B , Chen W , et al. Delayed help seeking behavior in dementia care: preliminary findings from the Clinical Pathway for Alzheimer’s Disease in China (CPAD) study. Int Psychogeriatr 2016; 28: 211–19. 10.1017/S1041610215000940 26138923

[21] Draper B , Cations M , White F , Trollor J , Loy C , Brodaty H , et al. Time to diagnosis in young-onset dementia and its determinants: the INSPIRED study. Int J Geriatr Psychiatry 2016; 31: 1217–24. 10.1002/gps.4430 26807846

[22] Fiske A , Gatz M , Aadnøy B , Pedersen NL. Assessing age of dementia onset: validity of informant reports. Alzheimer Dis Assoc Disord 2005; 19: 128–34. 10.1097/01.wad.0000174947.76968.74 16118529

[23] Abdel Aziz K , Sajwani HS , Galadari M , Al-Ammari A , AlHassani J , Al-Nuaimi N , et al. Psychiatric disorders in inpatient Ethiopian migrant workers in the United Arab Emirates. Transcult Psychiatry 2023; 60: 997–1004. 10.1177/13634615231200855 37753634

[24] World Health Organization. International Statistical Classification of Diseases and Related Health Problems. 10th Revision. WHO, 1992.

[25] Kaufer DI , Cummings JL , Ketchel P , Smith V , MacMillan A , Shelley T , et al. Validation of the NPI-Q, a brief clinical form of the Neuropsychiatric Inventory. J Neuropsychiatry Clin Neurosci 2000; 12: 233–9. 10.1176/jnp.12.2.233 11001602

[26] Faul F , Erdfelder E , Buchner A , Lang A-G. Statistical power analyses using G*Power 3.1: tests for correlation and regression analyses. Behav Res Methods 2009; 41: 1149–60. 10.3758/BRM.41.4.1149 19897823

[27] Van De Veen D , Bakker C , Peetoom K , Pijnenburg Y , Papma J , the PRECODE study group, et al. Provisional consensus on the nomenclature and operational definition of dementia at a young age, a Delphi study. Int J Geriat Psychiatry 2022; 37: gps.5691. 10.1002/gps.5691 PMC9305901 35156239

[28] Alhazzani AA , Alqahtani AM , Alqahtani MS , Alahmari TM , Zarbah AA. Public awareness, knowledge, and attitude toward Alzheimer’s disease in Aseer region, Saudi Arabia. Egypt J Neurol Psychiatry Neurosurg 2020; 56: 81.

[29] Hakami F , Madkhali MA , Saleh E , Ayoub R , Moafa S , Moafa A , et al. Awareness and perception toward Alzheimer’s disease among residents living in the Jazan Province, Saudi Arabia: a cross-sectional study. Cureus 2023; 15: e44505. 10.7759/cureus.44505 PMC10544552 37789999

[30] Benbow SM , Jolley D. Dementia: stigma and its effects. Neurodegen Dis Manage 2012; 2: 165–72.

[31] Zolezzi M , Alamri M , Shaar S , Rainkie D. Stigma associated with mental illness and its treatment in the Arab culture: a systematic review. Int J Soc Psychiatry 2018; 64: 597–609. 10.1177/0020764018789200 30019976

[32] Phung KTT , Chaaya M , Waldemar G , Atweh S , Asmar K , Ghusn H , et al. Validation of the 10/66 Dementia Research Group diagnostic assessment for dementia in Arabic: a study in Lebanon. J Geriatr Psychiatry Neurol 2014; 27: 282–90. 10.1177/0891988714532019 PMC4209200 24771602

[33] van Vliet D , de Vugt ME , Bakker C , Pijnenburg YAL , Vernooij-Dassen MJFJ , Koopmans RTCM , et al. Time to diagnosis in young-onset dementia as compared with late-onset dementia. Psychol Med 2013; 43: 423–32. 10.1017/S0033291712001122 22640548

[34] Nair SC , Sreedharan J , Satish KP , Ibrahim H. Health literacy in a high income Arab country: a nation-wide cross-sectional survey study. PLOS One 2022; 17: e0275579. 10.1371/journal.pone.0275579 PMC9534436 36197929

[35] Cova I , Markova A , Campini I , Grande G , Mariani C , Pomati S. Worldwide trends in the prevalence of dementia. J Neurol Sci 2017; 379: 259–60. 10.1016/j.jns.2017.06.030 28716255

[36] Bener A , Ghuloum S. Gender differences in the knowledge, attitude and practice towards mental health illness in a rapidly developing Arab society. Int J Soc Psychiatry 2011; 57: 480–6. 10.1177/0020764010374415 20591923

[37] Lee J , Lee KJ , Kim H. Gender differences in behavioral and psychological symptoms of patients with Alzheimer’s disease. Asian J Psychiatry 2017; 26: 124–8. 10.1016/j.ajp.2017.01.027 28483073

[38] Arsenault-Lapierre G , Henein M , Rojas-Rozo L , Sourial N , Bergman H , Couturier Y , et al. Primary care clinicians’ knowledge, attitudes, and practices concerning dementia: they are willing and need support. Can Fam Physician 2021; 67: 731–5. 10.46747/cfp.6710731 PMC8516166 34649895

[39] Arvanitakis Z , Shah RC , Bennett DA. Diagnosis and management of dementia: review. JAMA 2019; 322: 1589–99. 10.1001/jama.2019.4782 PMC7462122 31638686

[40] Walker Z , Possin KL , Boeve BF , Aarsland D. Lewy body dementias. Lancet 2015; 386: 1683–97. 10.1016/S0140-6736(15)00462-6 PMC5792067 26595642

[41] Outeiro TF , Koss DJ , Erskine D , Walker L , Kurzawa-Akanbi M , Burn D , et al. Dementia with Lewy bodies: an update and outlook. Mol Neurodegener 2019; 14: 5. 10.1186/s13024-019-0306-8 PMC6341685 30665447

[42] Al-Ramlawi DJ. Strategies of improvement of access to quality primary healthcare in the United Arab Emirates: lessons to learn. Smart Med J 2023; 6: 99.

[43] Kronfol NM. Access and barriers to health care delivery in Arab countries: a review. East Mediterr Health J 2012; 18: 1239–46. 10.26719/2012.18.12.1239 23301399

