# Supplementary material for: Determinants of time from symptom debut to dementia diagnosis in United Arab Emirates
Source: BJPsych Int. 2025 Nov 20;23(4):212–9. doi: 10.1192/bji.2025.10077 (PMC13732261; doi:10.1192/bji.2025.10077)
Supplement: Supplementary file 1 [file S2056474025100779sup002.pdf]

1. **Age at the time of dementia diagnosis**: _______

2. **Gender:** ☐ Male
   ☐ Female

3. **Nationality:** ☐ Emirati ☐ Other _________________

4. **Diagnosis:**

☐ Alzheimer's disease dementia	☐ Frontotemporal dementia
☐ Parkinson's disease dementia	☐ Vascular dementia
☐ Posterior cortical atrophy	☐ Lewy Body dementia
☐ Mixed dementia	☐ Other: ______________________

5. **Date of first contact with mental health services:** _____ Date _____ Month _____ Year

6. **Main symptom/s leading to initial presentation:**

☐ Forgetfulness	☐ Anxiety
☐ Delusions	☐ Elation/Euphoria
☐ Hallucinations	☐ Apathy/Indifference
☐ Agitation/Aggression	☐ Disinhibition
☐ Depression/Dysphoria	☐ Motor Disturbance
☐ Irritability/Lability	☐ Nighttime Behaviors
☐ Appetite/Eating	

7. **Cognitive examination/Score on initial presentation:**

☐ MMSE ______	☐ MoCA ______
☐ Others ______	

8. **When were the symptoms first noticed by family/patient?** ______ Month _____ Year

9. **Date of dementia diagnosis:** _____ Date _____ Month _____ Year
