# Supplementary material for: Determinants of time from symptom debut to dementia diagnosis in United Arab Emirates
Source: BJPsych Int. 2025 Nov 20;23(4):212–9. doi: 10.1192/bji.2025.10077 (PMC13732261; doi:10.1192/bji.2025.10077)
Supplement: Supplementary file 2 [file S2056474025100779sup003.docx]

# Supplementary Table S1. Sensitivity analysis of DUS with and without outliers

| Group | N | Mean | SD | Median | Q1 | Q3 | IQR |
| --- | --- | --- | --- | --- | --- | --- | --- |
| All cases | 825.0 | 34.65 | 28.83 | 24.0 | 12.0 | 48.0 | 36.0 |
| Excluding outliers | 822.0 | 34.18 | 27.76 | 24.0 | 12.0 | 48.0 | 36.0 |
