# Supplementary material for: Determinants of time from symptom debut to dementia diagnosis in United Arab Emirates
Source: BJPsych Int. 2025 Nov 20;23(4):212–9. doi: 10.1192/bji.2025.10077 (PMC13732261; doi:10.1192/bji.2025.10077)
Supplement: Supplementary file 3 [file S2056474025100779sup004.docx]

Supplementary Table S2. Multivariable linear regression analysis of DUS

| **Predictor** | **Estimate** | **SE** | **t** | **p** | **Standardised Estimate β** | **95% CI Lower** | **95% CI Upper** |
| --- | --- | --- | --- | --- | --- | --- | --- |
| Intercept | -22.421 | 6.7427 | -3.325 | < .001 | - | - | - |
| Age at diagnosis | 0.857 | 0.0823 | 10.417 | < .001 | 0.3299 | 0.268 | 0.3921 |
| Agitation/Aggression - [Initial symptoms: ref = Forgetfulness] | -21.726 | 2.844 | -7.639 | < .001 | -0.7537 | -0.947 | -0.56 |
| Depression/Dysphoria - [Initial symptoms: ref = Forgetfulness] | -10.308 | 4.483 | -2.299 | 0.022 | -0.3576 | -0.663 | -0.0523 |
| Delusions - [Initial symptoms: ref = Forgetfulness] | -21.545 | 7.809 | -2.759 | 0.006 | -0.7474 | -1.279 | -0.2156 |
| Irritability/Lability - [Initial symptoms: ref = Forgetfulness] | -17.11 | 3.807 | -4.494 | < .001 | -0.5936 | -0.853 | -0.3343 |
| Motor Disturbance - [Initial symptoms: ref = Forgetfulness] | -17.296 | 4.179 | -4.138 | < .001 | -0.6 | -0.885 | -0.3154 |
| Appetite/Eating - [Initial symptoms: ref = Forgetfulness] | -29.063 | 5.89 | -4.934 | < .001 | -1.0083 | -1.409 | -0.6072 |
| Hallucinations - [Initial symptoms: ref = Forgetfulness] | -33.595 | 4.693 | -7.159 | < .001 | -1.1655 | -1.485 | -0.8459 |
| Apathy/Indifference - [Initial symptoms: ref = Forgetfulness] | -1.481 | 8.993 | -0.165 | 0.869 | -0.0514 | -0.664 | 0.561 |
| Anxiety - [Initial symptoms: ref = Forgetfulness] | -23.663 | 8.984 | -2.634 | 0.009 | -0.8209 | -1.433 | -0.2091 |
| Nighttime Behaviours - [Initial symptoms: ref = Forgetfulness] | -20.679 | 5.816 | -3.556 | < .001 | -0.7174 | -1.113 | -0.3214 |
| Disinhibition - [Initial symptoms: ref = Forgetfulness] | -37.207 | 9.004 | -4.132 | < .001 | -1.2908 | -1.904 | -0.6776 |
| Female - [Gender: ref = Male] | -1.56 | 1.781 | -0.876 | 0.381 | -0.0541 | -0.175 | 0.0672 |
| Non-Emirati - [Nationality: ref = Emirati] | -0.373 | 1.857 | -0.201 | 0.841 | -0.0129 | -0.139 | 0.1135 |
| Vascular dementia - [Type of dementia: ref = Alzheimer] | -1.291 | 3.132 | -0.412 | 0.68 | -0.0448 | -0.258 | 0.1685 |
| Other - [Type of dementia: ref = Alzheimer] | -1.102 | 1.973 | -0.558 | 0.577 | -0.0382 | -0.173 | 0.0962 |
| Frontotemporal dementia - [Type of dementia: ref = Alzheimer] | 14.543 | 11.527 | 1.262 | 0.207 | 0.5045 | -0.28 | 1.2895 |
| Parkinson’s dementia - [Type of dementia: ref = Alzheimer] | -9.827 | 4.887 | -2.011 | 0.045 | -0.3409 | -0.674 | -0.0081 |
| Lewy Body dementia - [Type of dementia: ref = Alzheimer] | -8.398 | 13.238 | -0.634 | 0.526 | -0.2913 | -1.193 | 0.6101 |
| Mixed dementia - [Type of dementia: ref = Alzheimer] | -8.218 | 7.773 | -1.057 | 0.291 | -0.2851 | -0.814 | 0.2442 |
| Posterior cortical atrophy - [Type of dementia: ref = Alzheimer] | 30.032 | 18.074 | 1.662 | 0.097 | 1.0419 | -0.189 | 2.2726 |

Regression coefficients, standard errors (SE), t-values, p-values, standardised estimates (β), and 95% confidence intervals (CI) are shown.
